# Patterns of depression symptoms in relation to stressors and social behaviors during the COVID-19 pandemic among older youth and emerging adults in the United States

**DOI:** 10.1371/journal.pgph.0003545

**Published:** 2024-10-22

**Authors:** Kevin M. Cummins, Ty Brumback, Citlaly Corrales, Kate B. Nooner, Sandra A. Brown, Duncan B. Clark

**Affiliations:** 1 Department of Public Health, California State University, Fullerton, Fullerton, California, United States of America; 2 School of Psychology, Xavier University, Cincinnati, Ohio, United States of America; 3 Department of Psychology, University of North Carolina Wilmington, Wilmington, North Carolina, United States of America; 4 Departments of Psychology and Psychiatry, University of California San Diego, La Jolla, California, United States of America; 5 Department of Psychiatry and Pharmaceutical Sciences, University of Pittsburgh, Pittsburgh, Pennsylvania, United States of America; University of Ottawa Faculty of Medicine, CANADA

## Abstract

Substantial increases in depression at the outset of the pandemic were previously reported in NCANDA, a longitudinal sample of adolescents and young adults. The current NCANDA study examined depression symptoms before and during the COVID-19 pandemic. It evaluated the influence of stressors and social behavior (e.g., in-person and online socializing) with linear mixed effects models. A strong, positive association between COVID-19-related stressors and depression symptoms was observed. The frequency of in-person socializing did not account for the totality of the changes in depression observed during the early COVID-19 pandemic. It may be that pandemic-related stressors counteracted the benefits of in-person interactions during the early stages of the COVID-19 pandemic. Future studies can continue to elucidate the interactions among psychosocial, genetic, and behavioral factors contributing to depression symptoms in the unprecedented context of the COVID-19 pandemic.

## Introduction

Depression is one of the leading causes of disability, resulting in substantial social and economic burdens [1, 2]. Depression is also associated with reductions in quality of life [3] and excess mortality [4]. In addition, depression is a risk factor for suicidal behaviors [5], self-neglect [6], self-harm [7], and is a common co-occurring condition with substance use disorder [8, 9]. Although various pharmacological and nonpharmacological treatments have been developed and evaluated, the effectiveness of treatment-specific therapeutic components remains modest [10]. Depression remains a condition of public health concern.

Based on data from the US National Survey on Drug Use and Health, the prevalence of depression has been highest among adolescents (12–17 years) and young adults (aged 18–25 years) [11]. Depression’s prevalence has been increasing since 2012 in the United States and some other Western nations [11–14]. The prevalence has been consistently higher among females [15–17].

Depression among young people is of particular concern because it occurs during a developmental period of important transitions that influence future health, social, and economic outcomes [18, 19]. As a possible consequence of diminished academic functioning and attainment, poor health, and poor psychosocial functioning, young people with depressive symptoms are also more likely to experience unemployment in adulthood [20]. The total annual direct costs attributed to depression have reached $210 billion at the national level in the US [21]. Depression in early life may also result in sensitization to life stressors in later life that increase the risk of recurrence [22]. The current body of work on depression points to multiple interacting causes and vulnerabilities to depression ranging from prenatal exposures, childhood adversity, and exposure to parental mental disorder, to more proximal factors such as substance use, chronic pain, social factors, genetic liabilities, and psychosocial stressors [3, 23, 24].

The COVID-19 pandemic was associated with substantial declines in psychological well-being across multiple populations. During the early phase of the COVID-19 pandemic, elevated levels of distress and worsening mental health were reported in the US and countries around the world [25–27]. The prevalence of depressive symptoms increased by 300% during the first year of the pandemic, based on an early report by Xiong et al. [25]. Positive screenings for clinical depression and anxiety increased by 14% over the first year of the pandemic [28]. Later reports corroborated these findings [26, 27, 29, 30]. These increases were observed among various demographic groups, including older adolescents and emerging adults [26, 31–33]. The most pronounced increases in depression were observed among young women and girls [26, 32]. These increases coincided with changes to the psychosocial environment, particularly decreased in-person socializing.

Various aspects of social interactions are related to individuals’ well-being, including social network structure, network function, and perceptions of connectedness [34]. Large and diverse social networks appear to be protective against depression [34]. Evidence that perceived emotional support benefits well-being is substantial and appears to be more strongly associated with well-being than measures of actual instrumental support [34]. Among youth, the perception that social support is available has been found to be more strongly associated with decreased depression than received support [35]. However, the literature suggests better psychological outcomes are associated with more social capital, which may be partly due to instrumental resources available in the community and through strong and weak social relationships [36]. Generally, interactions with acquaintances are associated with more positive affect [37–39]. In comparison, social isolation is an established correlate of poor mental health outcomes [39–41].

During the early phase of the COVID-19 pandemic, numerous public health measures were introduced in the US and other countries to limit the transmission of SARS-COV-2, the virus that causes COVID-19. These non-pharmaceutical interventions (NPIs) included limiting in-person interactions through stay-at-home orders, switching school instruction to an online mode, restricting the operation of gathering places, banning indoor gatherings, and physical distancing practices [42, 43]. The NPIs disrupted the normal social interactions experienced by most youth in the US.

Rodman et al. [44] investigated social behaviors, perceived isolation, and perceived social support in the context of the COVID-19 pandemic among children and early adolescents (<16 years). Although perceived isolation from peers predicted internalizing and externalizing symptoms, no unconditional associations between in-person socializing and symptoms were detected. The difference between perceived isolation and in-person socializing findings demonstrates that perceived isolation is not a simple function of the quantity of social interactions. Consistent with other studies [45], Rodman et al. [44] found that pandemic-related stressors (e.g., health concerns, loss of income, etc.) predicted moderate increases in internalizing symptoms in children and, to a lesser extent, in early adolescents. For early adolescents, a concurrent association with stressors was observed only among those who reported reduced in-person social interactions [44].

Youth social interactions are not limited to in-person settings and frequently occur in online contexts, which NPIs did not impact. However, there are unique concerns regarding social media. Social media may function as a channel for adolescents to build social connections; nonetheless, some individuals who report higher levels of social anxiety and loneliness use social media more intensely. They experience little relief from their symptoms through social media use [46] and increased negative affect is associated with higher levels of social media use [47, 48]. Notably, several investigations early in the pandemic did not detect an overall change in patterns of digital interactions; high levels of digital socialization persisted with no evidence that change in online socializing was associated with increased depression symptoms in the population [44, 49, 50]. The changes in in-person or online social behavior have not been consistently identified as key mechanisms driving the substantial increase in depression symptoms in the community at the outset of the pandemic.

The overarching aim of the current study was to extend prior investigations into the relationship between social interaction and internalizing symptoms among older adolescents and emerging adults using a longitudinal sample established before the COVID-19 pandemic. This sample is the National Consortium on Alcohol and Neurodevelopment in Adolescence (NCANDA) [51]. Substantial increases in depression at the outset of the pandemic were previously reported for the NCANDA sample [26]; however, the correlates of these changes were not investigated. The first aim of the current study was to evaluate the extent to which the increases in depression at the onset of the pandemic were associated with in-person and online socializing. A second aim was to evaluate the extent to which depression symptoms were related to pandemic-related stressors. Given that sex-specific empirical findings related to depression are common [26, 32], each of the aims was analyzed with models with estimates of sex-specific patterns. The availability of the NCANDA study data spanning a period preceding and through the COVID-19 pandemic provides an unusual opportunity to evaluate the impacts of substantial stressors and social behavior on the healthy development of youth and young adults.

## Method

### Ethics statement

Institutional Review Boards (IRB) at each institution approved the study protocols (Duke University Health System IRB, SRI International IRB, University of California San Diego Human Research Protection Program, University of Pittsburgh IRB, Oregon Health Sciences University Research Integrity Office IRB). Adult participants provided written informed consent. Juveniles provided written informed assent in conjunction with written parental consent. The California State University, Fullerton IRB at the lead author’s institution also approved the secondary data analysis conducted in support of this study.

### Sample and procedure

Data for this study was obtained from NCANDA, an ongoing longitudinal panel study of health behaviors, psychosocial development, and neurocognitive development. The study uses an accelerated longitudinal cohort design (i.e., cohort sequential) [51]. Participants’ age range was ~9 years at any given wave of observations (12–21 years at baseline). Participants had been followed for up to 8 years at the outset of the COVID-19 pandemic. The participants were recruited from 5 different metropolitan catchment areas around each participating university: San Diego, California, at the University of California San Diego (UCSD); Durham, North Carolina, at Duke University; Portland, Oregon, at Oregon Health & Science University (OHSU); Pittsburgh, Pennsylvania at University of Pittsburgh; and Santa Clara County, California at SRI International. Recruitment was conducted to reflect the sex, racial, and ethnic distributions of the metropolitan region. Recruitment processes included public notices, random catchment-area direct phone calls, and blanket announcements distributed to entire student populations at local schools (see Brown et al., 2015 [51] for a detailed description of recruitment processes). By design, the sample ascertainment oversampled youth reporting a risk factor for alcohol use disorder, including alcohol use at an early age, internalizing or externalizing symptoms, and a family (parent) history of substance use disorder. About half of the sample (51%) had at least one risk factor at baseline [51]. Participant exclusion criteria at baseline included: current diagnosis with a severe psychiatric disorder (e.g., schizophrenia, bipolar disorder) that would interfere with achieving complete and valid assessments of the protocol, substance use disorder, current use of psychoactive medication, serious medical problems, intellectual disability or pervasive developmental disorder, lack of fluency in English, uncorrected sensory impairment, and prenatal drug or alcohol exposure of clinical significance.

The initial sample formed in 2015 consisted of 831 participants (S2 Table). Participants’ standard self-report assessment occurred annually via research assistant-administered clinical interviews and electronic self-report forms. A subset of participants also participated in weekly assessments of behavior via a brief (~1 minute) mobile app-based assessment [52] and two online assessments of participants’ experiences related to the COVID-19 pandemic [26]. Annual assessments were distributed across the year. The COVID-19 assessments occurred in waves that lasted several weeks. In total, 607 NCANDA participants provided data for the COVID-19 assessment, and 567 provided data from the three assessment protocols: annual visit, COVID-19 assessment, and mobile app assessment of social behavior. The COVID-19 assessment subset was described by Alzueta et al. [26]. The first COVID-19 assessments were issued between June 23, 2020, and July 10, 2020. The second was issued between December 7, 2020, and December 24, 2020. Four mobile app-based assessments during the study period included items related to social behavior. These occurred on April 13, April 20, and November 30, 2020, and March 8, 2021. Multiple community-wide NPIs for the COVID-19 pandemic were first instituted in March 2020 among the studied communities. The first COVID-19 assessment occurred approximately 80 days after substantial public health interventions were instituted in the US. The following assessment was issued approximately 266 days after interventions began.

### Measures

#### Demographics

Sex, age, race and ethnicity, family history of substance disorders and depression, and socioeconomic status were assessed during the staff-administered research interviews. Detailed information about the assessment protocol can be found in Brown et al. [51]. Youth socioeconomic status was approximated by parental education, given that parental contributions, loans, early career, or student status of the participants may obscure participants’ resources, environments, and social status [53, 54]. The highest educational attainment among parents was used in analyses as a marker of socioeconomic status. Detailed descriptions of the measures used in NCANDA and their administration are discussed in Brown et al. [51] and Cummins et al. [52].

#### Depression symptoms

The CES-D-10 was used to gauge the level of depression experienced by participants. The CES-D-10 is a validated version of the 20-item CES-D abbreviated to 10 items [55]. The CES-D-10 has demonstrated good psychometric properties in many communities and age groups, including adolescents and young adults [56]. Each item’s response options range from "rarely or none of the time" (coded as 0) to "all of the time" (coded as 3). A summative scale ranges from 0 to 30, with 0 indicating an absence of depression symptoms and higher scores for greater symptoms. Scores above ten are considered indicators of risk for clinical depression. The reference period for the symptom items is the prior week, which is short enough to capture state changes between assessments over the study period. The CES-D-10 was administered during annual interviews and the COVID-19 assessments.

#### Social behavior

Social behavior was assessed during weekly self-report via the mNCANDA mobile app. Weekly self-reports using the app randomly assessed multiple domains of behavior. Four assessments during the study period included items related to social behavior. One item asked participants to indicate on which of the past seven days they had socialized “in person (e.g., hanging out, recreating, dining, attending entertainment events),” and a second item asked which days they had been “Socializing online, where you were communicating with each other (e.g., texting, messaging, gaming with voice links).” The response options included individual checkboxes for each day of the week and ’none’ to allow participants to affirm they had not socialized. Assessments were issued on Monday mornings, and participants had the remainder of the day to complete the ~1-minute assessment. High response rates (>80%) were promoted through multiple reminder signals, a $2 flat rate incentive, and bonuses for consistent responding, resulting in up to $15 a month [52]. This incentive rate and the use of multiple reminders are similar to protocols used in other studies of youth [57] and were found to be acceptable by a substantial majority of the NCANDA sample [52].

#### COVID-19-related experiences

The survey instrument used in the two COVID-19 assessments was developed for the NCANDA study [26] and included 17 items related to COVID-19 impacts on employment, finances, housing, and education (see Supporting information). The list of impacts was designed to include factors expected to result in stress. Item responses were yes (coded as 1) or no (coded as 0); or not at all, slightly, moderately, very, extremely (coded as 0, 0.25, 0.5, 0.75, and 1, respectively). To gauge the breadth of COVID stressors experienced by participants, a composite score was created by computing the mean of the responses for the 17 items. Participants also reported if they or a family member had contracted COVID-19. The COVID-19-related items were issued as part of the two COVID assessments. Participants received $10 for responding to each of the online self-administered COVID assessments.

### Data analysis

#### COVID-19 base model (Model 1)

Mixed-effects models were used to model trajectories of depression symptoms over time (maximum of 10 observations per participant). In all the models in this report, the outcome variable was the CES-D-10 score. The marginal distribution was modeled as a left-censored normal distribution (i.e., Tobit regression; [58]. This model feature was included to address floor effects in the depression measure; 13.2% of the observations for CES-D-10 were zero, which is the minimum possible score. Fixed effects included sex and first and second-order quadratic terms for age. Age terms were interacted with sex to allow for sex-specific patterns to be estimated. Research site and parental education were entered as time-invariant covariates. Random effects were entered for participant intercepts. Because some participants had siblings in the study, a random effect was also included for families to address the non-independence of the siblings. Age was centered at the mean age of 19.5 years. In addition to these parameters, all models included an indicator variable for the COVID-19 pandemic timing: before or after the onset of the pandemic-related NPIs. The pandemic indicator variable interacted with sex and age so that changes in sex-specific age-based trajectories of depression were contingent on whether the pandemic had started. This paragraph describes the base model that all other models extended with additional parameters.

For subsequent models described below, the joint significance of differences in trajectory shapes as a function of specific exposure (e.g., socializing and stressors), sex, and the pandemic (i.e., their interactions with age) were evaluated with likelihood ratio tests of nested models [59]. Each model was compared to the base model with all the parameters and interaction terms except the exposure interactions. Where reported, main effects and average marginal parameter estimates were evaluated with Wald tests [59].

#### Social behavior models (Models 2, 3, 6 & 7)

Two sets of models were created to evaluate the associations between social behavior and depression (a maximum of 4 observations per case through the pandemic period). One model includes parameters for the number of days per week online socializing occurred (Model 2) and the other in-person socializing (Model 3). In each model, socializing was included as a main effect and interaction term with the trajectory parameters in the base model. Social behavior was included as a time-varying model parameter. The reports for social behavior were made on different dates than the depression assessments. The social behavior reports obtained proximally and prior to depression assessments were included in this study. The social behavior observations were temporally indexed with the subsequent depression assessments. In other words, the preceding social behavior reports were used as explanatory variables in the depression models. Although there may be bi-directionality in the causal relationship between social behavior and depression [60], the current models were developed to evaluate patterns that would be expected as a result of the potential effects of changes in social interactions following the instituting of NPIs. Models 6 and 7 then combine the social behavior with the COVID-19 stressors. These are described below.

#### COVID-19 stressors models (Models 4, 5, 6 & 7)

Two models were estimated to evaluate the association between COVID-19-specific stressors and depression trajectories. The first model incorporated a main effect and interactions for the variable that indicated if a participant or their family member contracted COVID-19 (Model 4). The second model incorporated a main effect and interactions for the COVID-19 stressors score with the base model (Model 5). Model 6 combined the stressors score model with the online socializing model, and Model 7 combined the stressors score model with the in-person socializing model.

#### Model estimation

The models were run on the subset of NCANDA participants who participated in the COVID-19-specific assessments (N = 655) and participated in the weekly mobile app-based assessment protocols (N = 568). A comparison of these subsets with the full sample demonstrated similar demographic characteristics with the full sample for the mobile app subset [52]. The COVID-19 assessment sample was likelier to be female, less likely to report being African American, and had lower socioeconomic status [26]. The intersection of these two subsets included 567 participants with a total of 4,315 observations. The participants were from 474 families. The mean number of observations before the pandemic was 4.9 (SD = 1.7). After the pandemic started, there was a mean of 3.1 observations (SD = 1.1).

The mixed-effects Tobit models used maximum likelihood estimation via mean-variance adaptive Gauss-Hermite quadrature with seven integration points. The Hessian matrix and gradient vector were computed with numerical approximations during optimization. All models converged. Marginal means (and 95% confidence intervals) estimated from each model were computed for pandemic period-specific trajectories across age by sex and exposures. Marginal effect sizes were reported on their original scale rather than standardizing them [61]. Variance estimates for marginal effects were computed using the delta method. Analyses were executed using the metobit command in Stata 16.1 [62].

Statistical significance was evaluated using a neo-Fisherian framework [63, 64], which considers P-values as providing a continuum of evidence [65]. Estimated parameter values (i.e., effect sizes) are reported as 95% confidence intervals or bands. These interval estimates were interpreted as providing plausible ranges for the parameter even when the estimate was not significantly different from the null value [66]. Model comparisons were aided by the comparison of Bayesian Information Criteria (BIC) and Akaike Information Criteria (AIC) [67]. BIC and AIC are comparative model fit statistics that penalize models for lack of parsimony (i.e., a larger number of parameters). Five-point differences are considered notable, and those with lower values are considered superior [67, 68]. Comparisons are made on models estimated with the same data. Where discrepancies in the patterns between AIC and BIC are encountered, deference is given to BIC as the selection of a model best representing the underlying mechanisms is sought rather than producing efficient prediction models [67, 69].

## Results

### Sample characteristics

At the baseline assessment, the mean age of participants in the sample was 16.1 years of age (range: 12–21) and was similar between the strata, with those ever reporting a CES-D-10 score (>10), the conventional cut point for considering a respondent at risk for depression [70], and those without (Table 1). The mean number of times participants were observed was 7.6 times, and the maximum number of times observed was 10. The mean age at the onset of the pandemic was 22.3 (SD = 2.54) years of age, with a range of 17 to 28 years. Females constituted 53.3% of the sample. In terms of self-reported race, most of the sample was white (72.7%), along with African American/Black (10.4%) and Asian (7.2%). A portion of the sample identified their ethnicity as Hispanic (11.6%). When stratified by CES-D-10 history, the percentage of each race in the sample varied by no more than four percentage points(Table 1). The proportion of Hispanics in the sample with a history of CES-D-10 scores above 10 was 53% higher than the lower CES-D-10 strata (Table 1). Parental education for the sample was high, with the majority (74.6%) having at least one parent having earned a college degree. Parental education in the sample was similar between strata (Table 1). The sample was relatively evenly distributed among five catchment areas, except San Diego, which had 27.5% of the sample. The distribution of elevated CES-D-10 scores varied across sites; rates of at-risk CES-D scores were elevated in the Portland and Palo Alto samples and lower in the Pittsburgh and Durham samples (Table 1).

**Table 1:** Sample demographics by depression history.

Variable	Depression History		Total
	Maximum CES-D < 10	Maximum CES-D ≥ 10	
n	237	330	567
Sex (% male)	53.6	41.8	46.7
Baseline Age (years)	16.1 (2.6)	16 (2.5)	16.1 (2.5)
Age at Pandemic (years)	22.37 (2.62)	22.27 (2.48)	22.31 (2.54)
**Race (%)**			
Native American	0.0	0.3	0.2
Asian	6.8	7.6	7.2
Pacific Islander	0.4	0.9	0.7
African American/Black	10.1	10.6	10.4
Caucasian/White	74.7	71.2	72.7
Other	8.0	9.4	8.8
Hispanic (%)	8.9	13.6	11.6
Parental Education (%)			
Less than High School	1.7	1.8	1.8
High School Graduate	11.8	13.9	13.1
College Graduate	75.1	74.2	74.6
**Site (%)**			
Pittsburgh, PA	18.1	14.8	16.2
Palo Alto, CA	16.5	21.2	19.2
Durham, NC	25.3	12.7	18.0
Portland, OR	13.1	23.3	19.0
San Diego, CA	27.0	27.9	27.5

### Descriptive statistics: Socializing patterns and stressors

**Fig 1:**
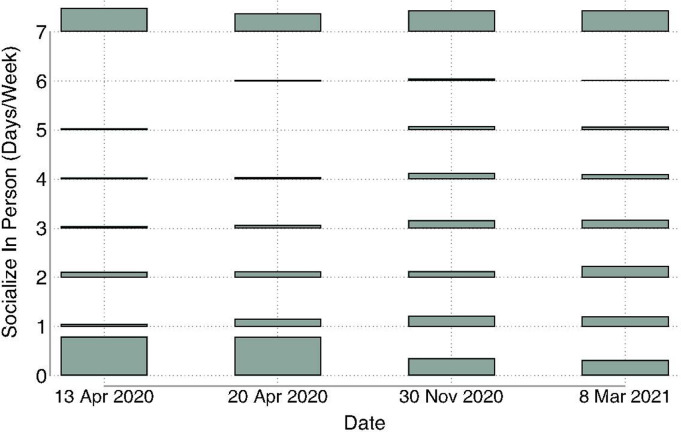
Distributions of in-person socializing frequency on four dates following the initiation of pandemic interventions. Bars represent the percentages on a given date. The maximum value was 51.1%.

**Fig 2:**
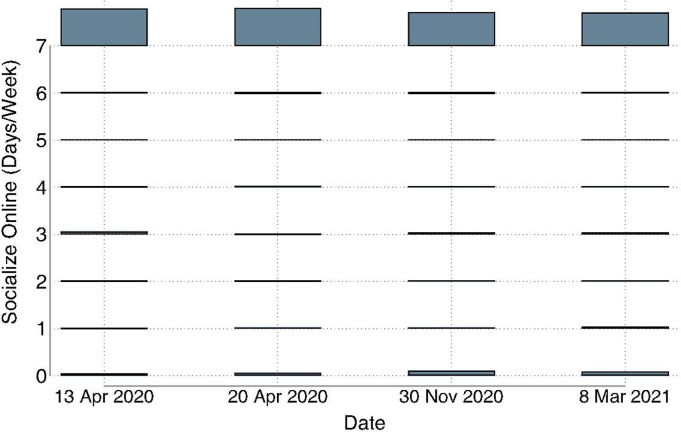
Distributions of online socializing frequency on four dates following the initiation of pandemic interventions. Bars represent the percentages on a given date. The maximum value was 84.0%.

During the first months of the pandemic, most of the sample reported not socializing in person (Fig 1). However, even at the earliest post-COVID onset assessment (43 days into the pandemic), 48.9% of the sample reported socializing in person at least one day a week (Fig 1) and >23% socialized seven days per week. By November 2020, in-person socializing dropped by 55.4% (Fig 1). During the start of the pandemic, the vast majority of the sample (>80%) reported socializing online daily (Fig 2). At the November 2020 assessment date, daily online socializing decreased slightly; the rate dropped to 75.1% (Fig 2).

Most participants reported at least one COVID-19-related stressor (90.1% for females; 87.2% for males). The mean number of these stressors was 6.0 (SD = 3.5) for females and 5.1 (SD = 3.4) for males. By the end of the study period, 43.0% of the sample had experienced a family member or close friend contracting COVID-19. The reports were higher among females (45.9%) than males (39.6%). No participants reported testing positive for COVID-19 at the first COVID assessment. By the second COVID assessment, 6.6% reported testing positive.

### Descriptive statistics: Depression patterns

The mean CES-D-10 scores remained between 4 and 5 during the six years before the pandemic for women in the sample (Table 2). Men’s scores were between 3 and 5 (Table 2). The mean and quartiles of CES-D mean scores were higher or equivalent for females compared to males (Table 2). CES-D-10 sample means increased (> 3 points) for both males and females at the onset of the pandemic. There was a doubling of the 75th percentile after the pandemic onset for both sexes in the sample (Table 2). After the pandemic onset, 39.3% and 37.4% of females and males, respectively, exceeded 10 points on the CES-D-10 scale, whereas less than 15% of the sample exceeded this threshold before the pandemic.

**Table 2:** CES-D-10 depression score and age summary statistics grouped by time and sex.

Assessment Years Relative to 2020										
	-8	-7	-6	-5	-4	-3	-2	-1	0	1
**Female**										
Mean (SD)	4 (3.49)	3.92 (3.7)	4.35 (3.91)	4.56 (4.45)	4.68 (4.29)	4.56 (4.93)	4.95 (4.98)	4.81 (4.64)	9.36 (6.44)	6.44 (5.14)
Median	3	3	3	3	4	3	3	4	8	5
P25, P75	1, 6	2, 5	1, 6	1, 7	1, 6	1, 6	1, 7	1, 7	4, 14	3, 8
**Male**										
Mean (SD)	2 (1.83)	3.55 (3.13)	3.41 (2.84)	3.58 (3.8)	3.75 (3.68)	4.11 (4.17)	4.31 (4.62)	4.67 (4.25)	7.66 (5.79)	4.82 (3.59)
Median	2	3	3	3	3	3	3	4	7	5
P25, P75	0.5, 3.5	2, 4.5	1, 5	1, 5	1, 5	1, 5.5	1, 6	2, 6	3, 12	2, 7
**Mean Age (SD)**	16.7 (1.6)	15.9 (2.5)	16.8 (2.6)	17.8 (2.5)	18.8 (2.5)	19.7 (2.4)	20.1 (2.3)	20.9 (2.3)	22.7 (2.5)	23.3 (2.6)

Note. P25 and P75 are the 25^th^ and 75^th^ percentiles, respectively.

### Depression trajectories

Overall, the strongest patterns that emerged from the models of CES-D-10 depression score trajectories were the sex differences and elevated scores after the onset of the pandemic. Associations between depression and in-person socializing were not discernable. For females, the association with online socializing was complex.

### Sex and pandemic (Model 1)

**Fig 3:**
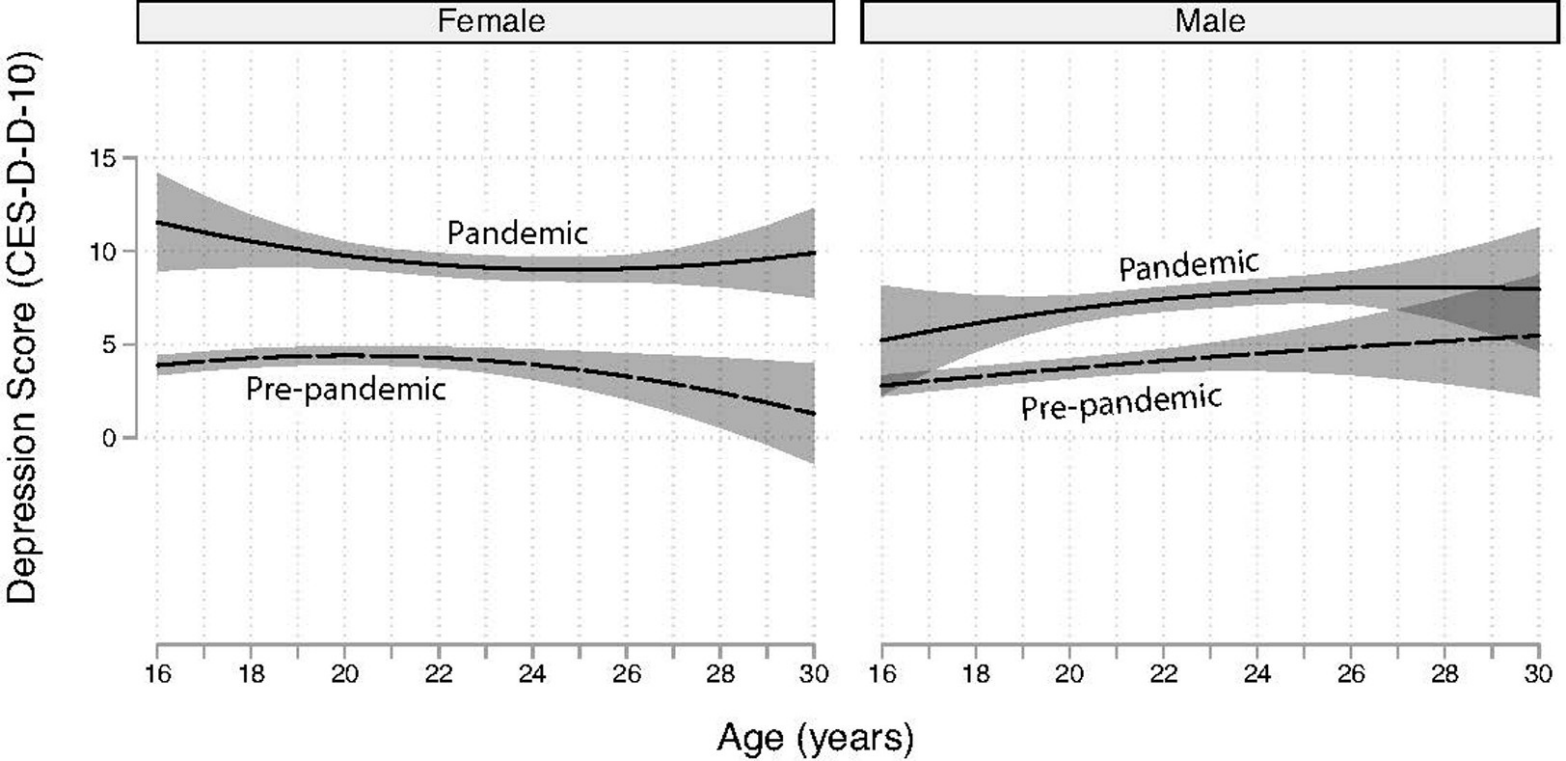
Modeled depression trajectories by sex and COVID-19 pandemic status based on Model 1 (base model). Marginal means and 95% confidence bands are shown. Dashed lines are CES-D-10 scores pre-pandemic and solid lines are during the pandemic.

The base model (Model 1) included sex-specific trajectories of CES-D-10 depression scores and the COVID-19 parameters. In this model, females had higher CES-D-10 scores than males (joint sex interaction test: LR X^2^ = 41.4, df = 6, P < .0001). During the pandemic, sex was the strongest predictor of the CES-D-10 (z = -4.1, P < .001 at age 22). In Model 1, females had 1.8 (95% CI [1.0, 2.3]) higher CES-D-10 scores than males during the pandemic at age 22. Before the pandemic, this difference was an order of magnitude lower (0.2; 95% CI [-0.7, 1.0]). The presence of different trajectory patterns by sex was consistent with subsequent models that included adjustments for stressors, family history of depression, and socializing frequency (Table 3, described in detail below). Pre-pandemic trajectories were relatively level up to age 22 with marginal mean CES-D-10 scores under 5 (Model 1, Table 3 and Fig 3). Estimates become less precise at older ages, which were under-sampled by design (Fig 3); however, there is weak evidence of a slight to moderate decline after age 25 among females during the pre-pandemic period that was less evident post-pandemic (Fig 3). Before the pandemic, males had lower mean CES-D-10 scores at age 16 but reach levels observed among females by late adolescence (Fig 3); depression scores among men increased linearly from 2.8 (95% CI [2.2, 3.3]) to 5.5 (95% CI [CI 2.2, 8.7]) between 16 and 30 years old; however, the estimation precision is highest below age 25 (Fig 3).

**Table 3:** Parameter estimates and model fit statistics for Tobit models of CES-D-10 depression scores.

*Model*	*Model 1*	*Model 2*	*Model 3*	*Model 4*	*Model 5*	*Model 6*	*Model 7*
*Variable*	Base	Online Socializing	In-person Socializing	Covid Diagnosis	Covid Stressors	Online X Stressors	In-person X Stressors
*Age*	0.02(0.05)	0.02(0.05)	0.02(0.05)	0.02(0.05)	0.02(0.05)	0.02(0.05)	0.02(0.05)
*Age2*	-0.03(0.01)**	-0.03(0.01)**	-0.03(0.01)**	-0.03(0.01)**	-0.03(0.01)**	-0.03(0.01)**	-0.03(0.01)**
*Sex (male)*	-0.8(0.36)*	-0.78(0.36)*	-0.77(0.36)*	-0.8(0.36)*	-0.79(0.36)*	-0.77(0.35)*	-0.78(0.36)*
*Sex* * *Age*	0.19(0.08)*	0.19(0.08)*	0.19(0.08)*	0.19(0.08)*	0.19(0.08)*	0.2(0.08)*	0.2(0.08)**
Sex * Age ^*2*^	0.03(0.02)	0.03(0.02)	0.03(0.02)	0.03(0.02)	0.03(0.02)	0.03(0.02)	0.03(0.02)
*Pandemic*	5.25(0.38)**	5.63(0.5)**	5.92(1.09)**	5.41(0.42)**	2.28(0.75)**	5.29(2.36)*	1.82(1.02)
Pandemic * Age	-0.4(0.2)*	-0.43(0.28)	1(0.65)	-0.57(0.23)*	-0.05(0.4)	-1.48(1.32)	0.22(0.56)
Pandemic * Age ^*2*^	0.07(0.03)**	0.07(0.04)	-0.18(0.08)*	0.09(0.03)**	0.06(0.05)	0.2(0.16)	0.04(0.07)
Sex * Pandemic	-2.33(0.55)**	-2.97(0.72)**	-2.39(1.51)	-2.58(0.61)**	-0.41(1.05)	-2.67(3.06)	0.18(1.38)
Sex * Pandemic * Age	0.56(0.31)	0.95(0.42)*	-0.88(0.93)	0.85(0.35)*	0.08(0.58)	-0.88(0.93)	-0.53(0.78)
Sex * Pandemic * Age ^*2*^	-0.1(0.04)*	-0.13(0.06)*	0.15(0.12)	-0.14(0.05)**	-0.09(0.08)	-0.37(0.23)	0(0.1)
*Site B*	1.12(0.56)*	1.12(0.56)*	1.07(0.56)	1.13(0.56)*	1.15(0.55)*	1.12(0.55)*	1.12(0.55)*
*Site C*	-0.77(0.57)	-0.76(0.57)	-0.77(0.57)	-0.77(0.57)	-0.69(0.56)	-0.68(0.56)	-0.7(0.56)
*Site D*	1.33(0.55)*	1.27(0.55)*	1.25(0.55)*	1.36(0.55)*	1.32(0.54)*	1.29(0.54)*	1.28(0.54)*
*Site E*	0.14(0.52)	0.18(0.52)	0.15(0.51)	0.18(0.52)	0.17(0.51)	0.21(0.5)	0.2(0.51)
*mParentEd*	-0.18(0.07)**	-0.19(0.07)**	-0.19(0.07)**	-0.18(0.07)**	0.17(0.51)	-0.19(0.07)**	-0.18(0.07)**
*Exposure*		-0.07(0.1)	-0.09(0.16)	-0.64(0.68)		-0.44(0.35)	0.2(0.22)
Exposure * Age		-0.02(0.06)	-0.25(0.1)*	0.58(0.4)		0.21(0.2)	-0.09(0.13)
Exposure * Age ^*2*^		0(0.01)**	0.04(0.01)	-0.07(0.05)		-0.02(0.03)	0(0.02)
Exposure * Sex		0.12(0.16)	-0.04(0.23)	1.03(1)		0.24(0.47)	-0.51(0.36)
Exposure * Sex * Age		-0.09(0.1)	-0.04(0.02)*	-0.97(0.63)		-0.28(0.27)	0.39(0.21)
Exposure * Sex * Age ^*2*^		0.01(0.01)	-0.04(0.02)*	0.15(0.08)		0.04(0.04)	-0.05(0.03)
*Stressors*					0.5(0.11)**	0(0.39)	0.63(0.15)**
Stressors*Age					-0.06(0.07)	0.55(0.24)*	-0.11(0.09)
Stressors*Age ^*2*^					0(0.01)	-0.08(0.03)**	0(0.01)
Stressors*Sex					-0.31(0.17)	0.21(0.6)	-0.52(0.22)*
Stressors*Sex*Age					0.08(0.1)	-0.69(0.36)	0.29(0.13)*
Stressors*Sex*Age ^*2*^					0(0.01)	0.12(0.05)	-0.03(0.02)
Exposure*Stressor						0.07(0.06)	-0.04(0.04)
Exposure*Age* Stressor						-0.1(0.04)**	0.01(0.02)
Exposure*Age ^*2*^ *Stressor						0.01(0)**	0(0)
Exposure*Stressor*Sex						-0.07(0.09)	0.11(0.06)
Exposure*Age*Stressor*Sex						0.12(0.05)*	-0.09(0.04)*
Exposure*Age ^*2*^ *Stressor*Sex						-0.02(0.01)**	0.01(0.01)*
*Constant*	7.11(1.23)**	7.15(1.23)**	7.23(1.23)**	7.07(1.23)**	7.05(1.21)**	7.21(1.2)**	7.07(1.21)**
*Family*	2.13(1.51)	2.33(1.51)	2.2(1.52)	2.18(1.51)	1.91(1.43)	1.91(1.44)	2.17(1.44)
*Person*	10.33(1.61)**	10.06(1.59)**	10.12(1.61)**	10.25(1.61)**	10.06(1.54)**	9.92(1.54)**	9.73(1.53)**
*Error*	15.46(0.39)**	15.21(0.39)**	15.15(0.39)**	15.45(0.39)**	15.14(0.38)**	14.78(0.38)**	14.86(0.38)**
*BIC*	22997.49	22256.97	22244.88	23043.03	22965.98	22255.42	22271.05
*AIC*	22870.09	22092.16	22080.07	22877.42	22800.37	22014.55	22030.18

Note. Exposure was the unique variable investigated in each model: online socializing (Models 2 & 6), in-person socializing (Models 3 & 7) family member diagnosed with COVID-19 (Model 4), COVID-19 stressors (Models 5, 6, & 7).

A substantial increase in depression scores was observed after the pandemic began in each of the models. The increases were significantly higher for females in the Base (Model 1), which was also observed in the subsequent Online Socializing (Model 2), In-person Socializing (Model 3), and Stressors Models (Model 5), (Table 3, P’s < .003; described below). The increase associated with the pandemic ranged from 5.7 CES-D-10 points (95% CI [4.8, 6.7]) for females and 3.0 CES-D-10 points (95% CI [2.0, 4.0]) for males at 19 years old, and 5.4 CES-D-10 points (95% CI [4.4, 6.3]) for females and 3.3 CES-D-10 points (95% CI [2.1, 4.4]) for men at age 25 years old in the Base Model. Estimates were poorly resolved at older ages where the interval estimates (i.e., 95% CI) of the pre-pandemic versus pandemic differences in depression scores broadly overlapped for males (Fig 3). For example, the interval estimate of the differences in depression scores pre- and post-pandemic onset ranged from 5.6 to 11.6 for females and -1.4 to 6.5 for males at age 30 years. The general patterns in the differences were similar across all models (Table 3).

### Online socializing (Model 2)

**Fig 4:**
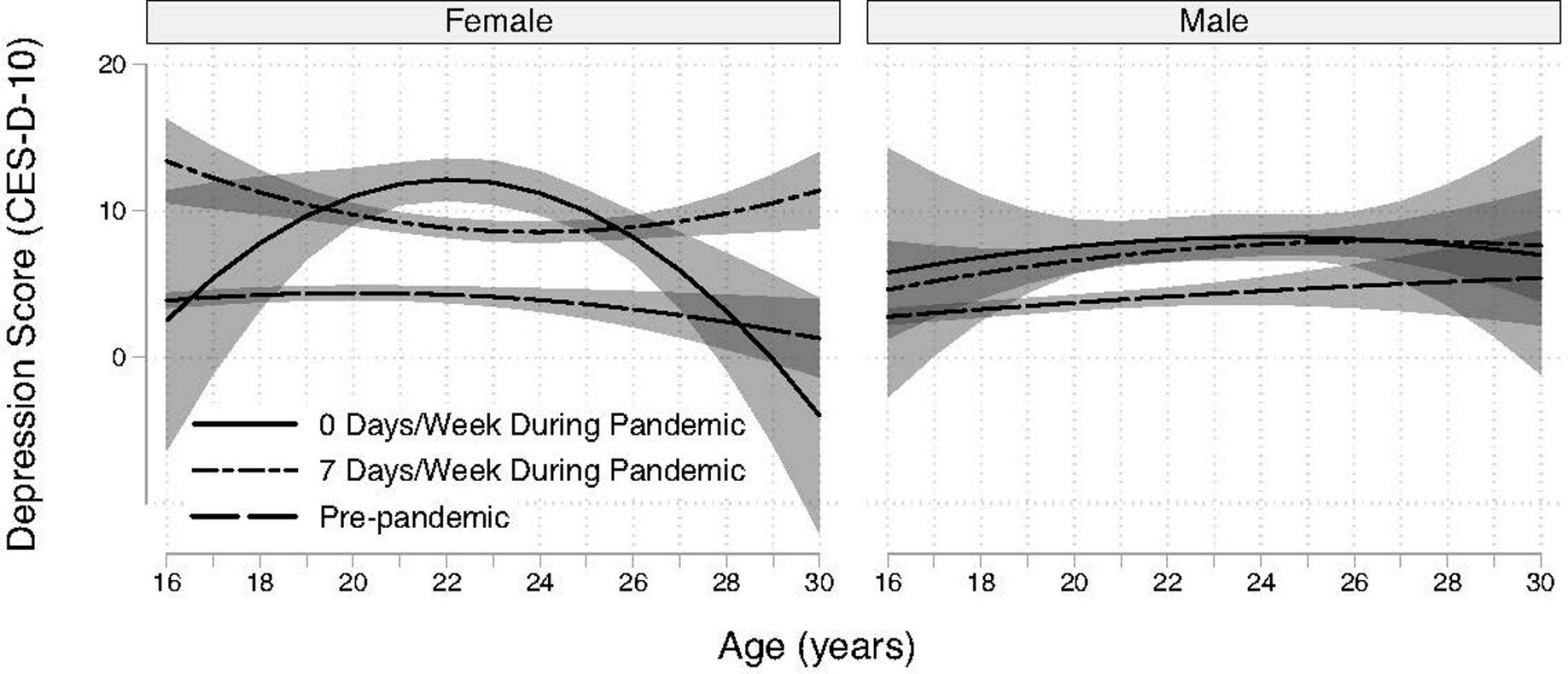
Modeled depression trajectories by sex, COVID-19 pandemic status, and frequency of online socializing based on Model 4 (online socializing model). Marginal means and 95% confidence bands are shown. Single dashed lines are depression scores pre-pandemic, solid lines are at the minimum online socializing frequency during the pandemic, and long-short dashed lines are at the maximum online socializing frequency during the pandemic.

**Fig 5:**
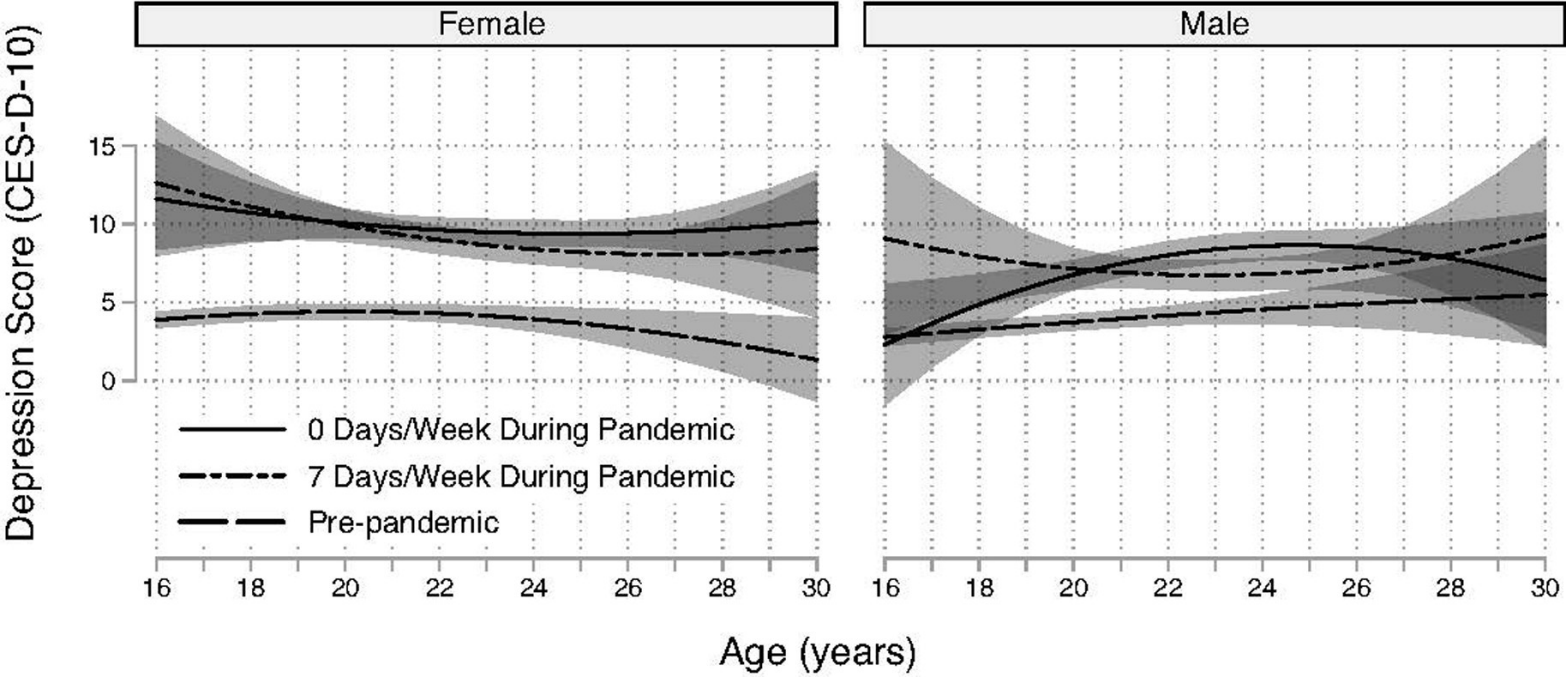
Modeled depression trajectories by sex, COVID-19 pandemic status, and frequency of in-person socializing based on Model 5 (in-person socializing model). Marginal means and 95% confidence bands are shown. Single dashed lines are depression scores pre-pandemic, solid lines are at the minimum online socializing frequency during the pandemic, and long-short dashed lines are at the maximum online socializing frequency during the pandemic.

The association between trajectories of depression scores and the frequency of online socializing was complex. The patterns of depression scores differed based on sex and frequency of online social interactions (LR X^2^ = 20.9, *df* = 6, P = .0019) and demonstrated age-related differences (Fig 4). Only among females was online socializing frequency found to be associated with depression score trajectories (Fig 4). Although only weakly significant (z = 2.29, P = .02), the modeled CES-D-10 score for females at 16 years was 10.9 points (95% CI [1.6, 20.2]) higher for those that socialized online every day as compared to those that did not report any online socializing. Between the ages of 21 and 24 years, depression scores were higher among females who reported less frequent online socializing online. For example, females aged 22 years reporting no online socializing had depression scores 3.31 points higher (95% CI [1.81, 4.81]) than those who socialized seven days a week (Model 2). Older females (> 27 years) with daily online socializing had substantially higher depression scores than those without any online socializing (Fig 5). Daily online socializing was associated with CES-D-10 scores above 10 points by age 30 (Fig 5). By age 30 years, females with no online socializing had depression CES-D-10 scores equivalent to or better than pre-pandemic levels (Fig 5).

### In-person socializing (Model 3)

An association between trajectories of depression scores and the frequency of socializing in person was not detected (Model 3; LR X^2^ = 11.6, df = 6, P = .07). Despite the lack of significance for socializing in person, the relatively narrow confidence bands at the younger ages do allow for the relative differences between the general pandemic effect and the range of plausible association magnitudes between socializing in person to be compared (Fig 5). The point estimate for the in-person socializing association during the pandemic (-0.09 CES-D-10 points/day of socializing in person/week) was 58 times smaller than the magnitude of the association with the pandemic onset (Table 3). Even when considering the difference between *daily* in-person socializing (7 days/week), the association was eight times smaller than that seen with the pandemic onset.

Particularly for females, the confidence bands for the marginal means predicted by the model at the extremes of socializing rates (none and daily) broadly overlapped (Fig 5). The *point estimates* for CES-D-10 scores during the pandemic were lower with less in-person socializing among males under age 20 years and older than 28 years (Fig 5). Between 20 and 28 years the *points estimates* for CES-D-10 scores are negatively associated in-person socializing among males. At age 22, the confidence interval ranged from -0.25 to 0.07 (z = -1.1, P = .27) for females and from -.35 to -.003 (z = -2.0, P = .046) for males. In summary, the overall differences in trajectories by sex, pandemic status, and level of in-person socializing were not discernible from a null model. Still, the interval estimates demonstrate that most of the pandemic-related increase in depression is not attributable to in-person socializing in Model 3.

### Stressors (Models 4 & 5)

The COVID-related stressors models were among the least superior models based on BIC (Models 4 & 5 in Table 3). Although the model with the COVID-related stressor score (Model 5) had a BIC 31.51 points lower than the Base Model (Model 1), it was also substantially higher (> 709 points) than the socializing models (Models 2 and 3). The COVID diagnosis model was unremarkable and did not improve estimation of the exposure variables. Models that included socializing frequency were superior to the Base and COVID-related stress models based on BIC and AIC (Table 3). Using the 5-point benchmark for differences in BIC, the in-person socializing model was superior to all other models.

The interaction of COVID-related stressors with the sex-specific trajectories (Model 5) was far from significant (LR X^2^ = 8.3, df = 5, P = .14). Although no changes in the shapes of the trajectories as a function of the stressors were detected, a first-order association between stressors and CES-D-10 scores was detected (Table 3). The marginal mean was 0.45 CES-D-10 points higher per stressor (95% CI [0.075, 0.82]; z = 2.4, P = .02) among females; thus, the model predicts a 7.6 (95% CI [1.27, 13.9]) point rise for females that experienced all the COVID-related stressors (stressors score = 17). For males, the association with stressors was unresolved, with a point estimate for the slope of 4.9 points/17 stressors (95% CI [-2.4, 12.2]; z = 1.3, P = .19).

### Stressors X socializing frequency (Models 6 & 7)

Modification of the relationships between the sex-specific and socialization rate-based CES-D-10 trajectories by stressors (i.e., socializing frequency X sex interaction X stressors) was moderately significant in Model 6 (Stress & Online Socializing Model) and Model 7 (Stress & In-person Socializing Model); the significance of the stress interaction terms as a group in the Stress & Online Socializing Model was P = .02 (Model 6; LR X^2^ = 21.8, *df* = 11). The significance of the stress interaction terms as a group for the Stress & In-person Socializing Model was slightly lower (Model 7; LR X^2^ = 24.9, *df* = 11, P = .01). Between stress by socializing interaction models (Models 6 & 7), the online socializing model had superior information criteria statistics in comparison to the in-person model. However, all of the models with stressors (Models 4, 5, 6 & 7) were inferior to the In-person Socializing Model without stressors (Model 3) based on BIC (Table 3). In summary, moderation by stressors was relatively weak or poorly resolved.

Key exemplars are provided to provide examples of the estimated associations at different levels of socializing and stress in Models 6 and 7. At age 25, each additional stressor was associated with .32 (95% CI [0.07, 0.58]) and .65 (95% CI [0.33, .98]) higher CES-D-10 scores at no days socializing in person and every day socializing online, respectively, for females. In other words, the *point estimates* are such that the increase in the level of depression associated with stress doubled when comparing no socializing in person with daily socializing in person increases. However, the interval estimates for the association substantially overlap across the range of online socializing frequency. Estimates at older ages are similar but even less precise. On the other hand, at younger ages, the relative changes in point estimates for the stressor effects reverse, and the confidence intervals expand. For example, at no days socializing in person and daily socializing in person, each additional stressor was associated with 0.78 (95% CI [0.22, 1.35]) and -.15 (95% CI [-0.83, 0.53]) for females at age 19 years, respectively. Again, the interval estimates overlap. The same set of estimates for males at age 25 years is 0.66 (95% CI [.32, 1.00]) and 0.63 (95% CI [0.26, 1.00]); at 19 years for males, the estimates are -0.25 (95% CI [-0.79, 0.28]) and 0.98 (95% CI [-0.14, 2.11]).

The patterns of online socialization were even less resolved. For females at age 25, the stressors were associated with .52 (95% CI [0.06, 0.99]) and .46 (95% CI [0.23, 0.69]) points higher CES-D-10 scores at no days socializing online and everyday socializing online, respectively. For females at age 19, stressors were associated with -0.03 (95% CI [-1.55, 1.48]) and .42 (95% CI [-0.04, 0.88]) higher scores at no days socializing in person and daily socializing in person, respectively. Among females, the *point estimates* of the stressor association were similar at older ages regardless of online socialization frequency. In contrast, the *point estimates* were positive at younger ages only for those socializing online. However, the interval estimates for the effects overlap broadly in all cases. The pattern is similar for males, although the estimation precision is generally much lower (standard errors reach 2.33 for the marginal estimates between 16 and 25 years).

### Summary

The strongest associations identified were between the pandemic and depression among both sexes. Other patterns were weak or less resolved, with several exceptions. For example, an association between the COVID-stressors score and depression was identified among females. The association for males was not resolved. Associations with socializing behavior were most discernable for online socializing, with greater depression observed with no online socializing among women in their mid-twenties.

## Discussion

The current report is based on data from NCANDA, a longitudinal study of youth and young adult neurodevelopment initiated before the COVID-19 pandemic. This study took advantage of a well-characterized large sample with up to eight years of repeated measures of depression prior to the pandemic [26, 71–73]. In addition, the results are based on data from an established panel that is demographically representative of youth in the metropolitan regions around the five participating research universities [51], with the likely exception of the underrepresentation of low socioeconomic status families in the sample. While some of this study is exploratory, it also includes confirmatory aspects, which is a strength. The models with the greatest complexity were structured to align with the previously reported findings by Rodman et al. [44].

### COVID-19 pandemic and sex

The prevalence and intensity of depression abruptly increased among populations worldwide during the early phase of the COVID-19 pandemic [26, 74–78]. The upsurge at the start of the pandemic followed years of gradual escalation of depression [79], particularly among adolescents and young adults [12, 80]. The current study’s findings are consistent with these patterns.

Alzueta et al. [26] found that the risk of depression tripled in the NCANDA sample at the outset of the COVID-19 pandemic, with females being at greatest risk. The current analysis also demonstrates a dramatic increase in depression scores beyond what was expected during normal development. Depression among young adults appeared to have increased by at least 4.8 and 2.0 CES-D-10 points for females and males, respectively. This increase is substantial, considering that the 75th percentile was generally around 7 points for females and 6 points for males before the pandemic (Table 2). Whereas Alzueta et al. [26] modeled the conditional CES-D-10 scores with a Poisson distribution, the current study employed a left-censored Tobit regression to address the floor and semi-continuous depression scale based on the sum of coded responses for the ten ordinal response items. The overall findings between the earlier and current reports are structurally consistent despite the different modeling approaches. The current study extends prior work on youth and young adult mental health during the pandemic by evaluating the correlates of depression trajectory patterns, including the frequency of social interactions and pandemic-related stressors, and employing an alternative analytic approach.

### Social behavior

Engagement with social media is suspected of being one of multiple factors that explain the multi-decade trend of increasing depression symptoms among youth in the US [12, 81]. The earliest assessment of socializing patterns occurred in the second month of the pandemic when US national public health authorities recommended NPIs such as social distancing and closing venues where people gather [42, 43]. Schools had moved from in-person settings to online. Typical patterns of social interaction were disrupted. Despite the NPIs, half of the young people in this study reported socializing in person at least once a week early in the pandemic. About a quarter did so daily. Daily online socializing was common throughout the pandemic.

The variability in socializing provided the opportunity to evaluate the relationship between social interactions and depression in a situation where there was an exogenous driver of reduced in-person interactions. The strongest patterns identified in the current study related to social behavior were for online socializing (Fig 5). The patterns were complicated. For younger females, less online socializing was associated with greater depression. The opposite was the case for those older than 26 years, and by age 30, those who didn’t socialize online had depression scores similar to people of the same age before the pandemic. Among males, no detectable patterns between online socializing frequency and depression were found. The inconsistent patterns among ages and between sexes make it challenging to compare with Rodman et al.’s [44] findings on social interactions and internalizing symptoms among older children (7-10y) and young adolescents (13-15y), as their published analyses did not allow for sex-specific patterns to be investigated.

This study’s results for in-person socializing are easier to compare to the Rodman et al. (2022) findings. They did not detect a first-order association between in-person socializing and internalizing symptoms. This finding is consistent with the current findings, wherein the association between in-person socializing frequency and depression symptoms was only detectable when trajectories were contingent on the level of COVID-19-related stressors experienced by the participant. Although the patterns of the In-person Socializing Model could not be resolved, the depression patterns for males between ages 22 and 25 years were consistent with either a null pattern or with higher levels of in-person socializing being only partially protective against pandemic-associated increases in depression. If in-person socializing were fully protective, the magnitude of the association would be expected to reach the depression increase associated with the pandemic onset. The interval estimates for 18- to 26-year-olds demonstrate that, at most, only part of the pandemic effect is explained by in-person socializing; this occurs within the age range where the models’ precision (i.e., statistical power) was greatest. Although all the patterns could not be fully resolved with the statistical power provided by the NCANDA sample, the findings do provide boundaries on the likely magnitude of association [66]. Taken together, the results suggest that the pandemic-related rise in depression cannot be fully attributed to reductions in the frequency of in-person socializing. The findings are also compatible with in-person socializing during the early part of the pandemic, having no association for either sex. In sum, in-person social interactions do not appear to provide a full explanation for the depression increases observed among young people.

The findings would be surprising if all in-person interactions were equivalent and exchangeable. They are not. While many constructs that characterize or emerge from patterns of social interactions are well-established as strong correlates of well-being and depression, they don’t all operate in the same manner [34]. These constructs include loneliness, perceived isolation, emotional support, instrumental support, connectedness, and negative interactions [82–86]. Some of these emerge from a history of interactions with social counterparts and are not a direct function of the recent frequency or duration of interactions.

Further, in-person social interactions are not always beneficial. Negative in-person interactions may exacerbate negative affect and cognitions. Negative social interactions have been shown to cause depression-like behaviors in experimental studies of animal models [87]. As a whole, the mechanisms involved in the effects of short-term changes of in-person socializing are complex. That complexity was not measured. This challenge of capturing social complexity is a key limitation of the current study and much of the related literature.

Another challenge to our understanding of the relationship between social-related constructs and depression is that persons with depression are more likely to retract from social interactions, potentially limiting social networks as a source of support and connectedness. Social withdrawal is a characteristic and consequence of mental health disorders, including depression [88, 89]. In many instances, intentional social withdrawal precedes the manifestation of many diagnostic features of depression [88]. Thus, an important strength of the current study is that an exogenous event, the pandemic, triggered many of the alterations in social behavior that were initially observed.

From a public health standpoint, considering the impacts of NPIs is a reasonable early step in the investigation of social behaviors and their correlates. In-person social interactions are the behavior most relevant to the transmission of the virus that causes COVID-19. Although the assessment of social behavior used in this study was based on self-report, the assessments were repeated and limited to the prior seven days. This short assessment period allowed participants to enumerate the frequency of the behavior and potentially lower recall bias. The characteristics and diversity of social interactions are not addressed in the current study’s assessment of social interactions. This limitation was noted above. The potential impact of this limitation could be addressed in future studies investigating the mechanisms behind the age- and sex-related differences observed in the present study. Doing so would be an important step in building a body of work that can help inform policy and intervention development.

### Stressors

Determinants of depression include many factors such as early life adversity, neighborhood and family environment, genetics, and stressors, including social stressors [19, 90–92]. Such factors were not incorporated into the present analyses and are missing in much of the current COVID-19-related literature. This gap may be addressed in future analyses of NCANDA data as genetic and family history assessments are underway. The current analyses are somewhat constrained without the ability to characterize who in the sample were most vulnerable because of their genetics. It is possible that associations may be substantial only for those with pre-existing vulnerabilities.

The positive association between COVID-19-related stressors and depression symptoms was one of the strongest patterns identified in the current study. COVID-19-related stressors were also a strong predictor in Rodman et al.’s [44] models of internalizing symptoms. This finding demonstrates that associations were detectable and consistent with expectations despite this study’s limitations, including using a simple composite summary score to measure exposure to stress. All stressors are treated as equivalent for each participant. However, the psychological impacts of each type of stressor likely vary. In addition, only COVID-19-related stressors were addressed here. Thus, the current analyses likely underestimate the role of stress. The study may have also missed capturing the effects of some economic stressors that were more common among lower socioeconomic families because the study sample came from families with relatively high college education rates. Thus, the conclusions may not generalize to families with lower educational attainment.

Not measured in the current study or by Rodman et al. [44] were potentially severe stressors associated with reactance and community affiliation that can arise in response to disasters or national crises. During the pandemic, negative cognitions and affect in response to a threat to personal freedoms (i.e., reactance) were greatest among those who perceived the health threat of COVID to be less important when subject to public health policies and information [93]. In turn, those with greater reactance were less likely to adhere to NPIs related to limiting in-person interaction [93]. Some young adults participating in high-frequency in-person socializing were more likely to have been among those experiencing pandemic-related-reactance stresses not commonly assessed in studies of youth mental health. Conversely, some young adults limiting in-person social interactions may have done so as part of their sense of affiliation with the broader community. Group affiliation and the shared purpose in addressing the pandemic together appeared to be protective against depression [94]. This community factor might partially explain why greater adherence to NPIs was associated with lower depression levels, particularly for women and people with close relationships with vulnerable people [77]. These complexities could have blunted the ability to detect the isolated unconditional effects of in-person socializing in this study.

### Stressors and social behavior

Although an unconditional association between in-person socializing and trajectories of internalizing symptoms was not detected, conditional associations were detected by Rodman et al. [44] and in the current study. Because social support is associated with reduced vulnerability to stressors [95], the rate of socializing behaviors was added as a moderator of the COVID-19 stressors in models of mental health outcomes in both studies. In the current study, modification of the sex- and socializing-level-specific trajectories by COVID stressors was explored in two models (Models 6 & 7). Unsurprisingly, the precision of the estimates in these models was low, given the large numbers of estimated model parameters and the limitations mentioned above. Although the sample is relatively large for a longitudinal study of neurodevelopment, resolution in these complex models was limited, particularly for age ranges with fewer observations. The models included fully crossed interactions for sex, pandemic, socializing frequency, stressors, and two parameters for age. These complex models were necessary to address the research aims and evaluate the extent to which the patterns reported by Rodman et al. [44] were replicated in the NCANDA study. Rodman et al. [44] reported that more social interaction was associated with lower internalizing symptoms in the face of COVID-related stressors. Rodman et al. [44] concluded that socializing might be protective against the depression-triggering effects of pandemic-related stressors. However, the findings of this study do not support the frequency of in-person socializing with friends as a dominant instrumental determinant of depression symptoms for older adolescents and young adults.

## Conclusion

The current report contributes to a body of work investigating how the stressful impacts of the pandemic and two types of social behavior during the COVID-19 pandemic related to changes in depression symptoms for adolescents and young adults. Stressors unique to the pandemic were associated with greater depressive symptoms. In contrast, the frequency of in-person socializing did not account for most of the elevation in depression observed among older adolescents and young adults during the early phase of the COVID-19 pandemic. The relatively weak effects of social interactions may be surprising, considering that randomized social isolation experiments with primates and rodents demonstrate that the loss of social interactions causes neurobiological and behavioral effects analogous to human depression [96–100]. Unlike the mammals in those experiments, the young adults in this study were not entirely isolated. Many had daily in-person social interactions with friends. Most also had daily online interactions. The online interactions complicate the conclusions made here. Both face-to-face and phone/video contact with another person are associated with lower depression among adults [83]. Unlike text-based communications, video and voice interactions allow for exchanging real-time non-verbal signals and cues. The signals and cues may help to maintain and enhance a sense of connectedness and emotional support that existed before the pandemic onset. Unfortunately, the current study’s measure of online socializing did not distinguish between textual and phone/video interactions. Some participants’ online interactions may have been a better substitute for in-person interactions than those experienced by their peers.

Our findings may indicate that additional factors unrelated to socializing contributed to the increase in depression symptoms following the onset of the pandemic. It may be that the milieu of pandemic-related stressors offset some benefits of in-person interactions or enhanced factors such as reactance to NPIs.

## Supporting information

S1 Table Site-specific recruitment periods. (DOCX)

S2 Table Site-specific recruitment periods. (DOCX)
