# Supplementary material for: Patterns of depression symptoms in relation to stressors and social behaviors during the COVID-19 pandemic among older youth and emerging adults in the United States
Source: PLOS Glob Public Health. 2024 Oct 22;4(10):e0003545. doi: 10.1371/journal.pgph.0003545 (PMC11495575; doi:10.1371/journal.pgph.0003545)
Supplement: S1 Table — (DOCX) [file pgph.0003545.s001.docx]

S1 Table. Site-specific recruitment periods.

| University | Location | Date First Subject Enrolled | Date Last Subject Enrolled |
| --- | --- | --- | --- |
| University of Pittsburgh | Pittsburgh, PA, USA | 07-FEB-2013 | 30-JUN-2014 |
| SRI | Palo Alto, CA, USA | 22-JAN-2013 | 23-SEP-2014 |
| Duke University | Durham, NC, USA | 18-JAN-2013 | 02-OCT-2014 |
| Oregon Health & Science University | Portland, OR, USA | 27-FEB-2013 | 28-JUN-2014 |
| University of California San Diego | San Diego, CA, USA | 19-NOV-2012 | 29-AUG-2014 |
